# Sample Size Considerations for One-to-One Animal Transmission Studies of the Influenza A Viruses

**DOI:** 10.1371/journal.pone.0055358

**Published:** 2013-01-31

**Authors:** Hiroshi Nishiura, Hui-Ling Yen, Benjamin J. Cowling

**Affiliations:** 1 School of Public Health, The University of Hong Kong, Hong Kong SAR, China; 2 PRESTO, Japan Science and Technology Agency, Saitama, Japan; 3 Centre of Influenza Research, School of Public Health, Li Ka Shing Faculty of Medicine, The University of Hong Kong, Hong Kong SAR, China; University of Oxford, Viet Nam

## Abstract

**Background:**

Animal transmission studies can provide important insights into host, viral and environmental factors affecting transmission of viruses including influenza A. The basic unit of analysis in typical animal transmission experiments is the presence or absence of transmission from an infectious animal to a susceptible animal. In studies comparing two groups (e.g. two host genetic variants, two virus strains, or two arrangements of animal cages), differences between groups are evaluated by comparing the proportion of pairs with successful transmission in each group. The present study aimed to discuss the significance and power to estimate transmissibility and identify differences in the transmissibility based on one-to-one trials. The analyses are illustrated on transmission studies of influenza A viruses in the ferret model.

**Methodology/Principal Findings:**

Employing the stochastic general epidemic model, the basic reproduction number, *R*_0_, is derived from the final state of an epidemic and is related to the probability of successful transmission during each one-to-one trial. In studies to estimate transmissibility, we show that 3 pairs of infectious/susceptible animals cannot demonstrate a significantly higher transmissibility than *R*_0_ = 1, even if infection occurs in all three pairs. In comparisons between two groups, at least 4 pairs of infectious/susceptible animals are required in each group to ensure high power to identify significant differences in transmissibility between the groups.

**Conclusions:**

These results inform the appropriate sample sizes for animal transmission experiments, while relating the observed proportion of infected pairs to *R*_0_, an interpretable epidemiological measure of transmissibility. In addition to the hypothesis testing results, the wide confidence intervals of *R*_0_ with small sample sizes also imply that the objective demonstration of difference or similarity should rest on firmly calculated sample size.

## Introduction

The transmission potential of a respiratory virus is commonly measured by the basic reproduction number, *R*_0_, i.e. the average number of secondary cases produced by a typical primary case throughout the entire course of host infection, which has been regarded as one of the most important quantities in infectious disease epidemiology [1]. The value of *R*_0_ not only informs how transmissible an infected individual is, but also gives three epidemiological insights into the transmission dynamics, i.e., (i) the risk of observing an epidemic given a certain number of infected individuals, (ii) the risk of infection in an individual throughout the course of an epidemic (given the epidemic), and (iii) the minimum control effort that is required to prevent or curb an epidemic based on a threshold theorem [1]. Among various methods for estimating *R*_0_, animal transmission experiments have been used as a useful tool for measuring the transmissibility in controlled conditions [2], although it should be noted that *R*_0_ is not only the property of a pathogen but also reflects the specific entire host-pathogen-environmental system, and thus, *R*_0_ in the experimental setting is not directly applicable to other (e.g. natural) settings. However, through transmission experiments, one can identify the importance of various factors on transmissibility, including host (e.g. genetic variants or immune status), virus (e.g. different strains), and environmental (e.g. ambient temperature/humidity) factors and their interactions, thereby providing important insights into mechanisms of infection and transmission.

Transmission experiments of influenza A viruses have helped to determine the molecular mechanisms of adaptation in human host due to the multi-host nature of the virus [3]–[5]. It is possible to study transmission in humans in controlled experimental settings [6] and natural settings [7], but such studies are resource intensive. Moreover, prior influenza exposure history in humans varies between individuals and is difficult to control. Although animal studies cannot replace human studies, they can provide complementary information on factors affecting transmission. The ferret model tends to be preferred over the mouse and guinea pig models for transmission experiments, because ferrets display clinical signs and symptoms which include fever, nasal discharge, and sneeze reflex [8]–[10]. In some studies, the potential of respiratory droplet transmission (inclusive of both droplet and aerosol transmissions) has been examined by placing a susceptible ferret in a cage next to another cage with a ferret inoculated with an influenza virus and allowing an exchange of air between the cages. Typically, such one-to-one transmission has been examined for two to four pairs for each virus, and the proportion of pairs with successful transmission has been compared between two (or more) different influenza viruses.

Despite a number of published ferret transmission experiment studies of influenza viruses, there has been no explicit estimation of *R*_0_ from experiments that typically involve very small sample sizes, and moreover, the sample size rationale of such an experiment has not been extensively discussed. Although a common approach is now to use three pairs of ferrets in each virus group, and to compare the proportions of pairs with successful transmission between each group, the appropriate sample size and proper analysis of results have not been investigated. For instance, suppose that researchers did not observe any transmission for one virus (i.e. *k*/*n* = 0/3 where *n* and *k* represent the numbers of pairs and infected pairs, respectively), while all three pairs resulted in transmission for the other virus (i.e. *k*/*n* = 3/3). Given this result, we would like to know (i) if the difference in the transmissibility between the two groups is statistically significant and (ii) the degree of difference in transmissibility given the small sample size. The present study aimed to discuss the significance and power of one-to-one animal transmission experiments with particular reference to ferret transmission studies of influenza A viruses as a case study.

## Materials and Methods

### One-to-one transmission experiment data

We start by presenting summary results of the published transmission experiment studies of influenza A viruses using the ferret model. Table 1 summarizes a total of 12 transmission experiment studies that were conducted under a one-to-one transmission experiment design [3], [5], [8], [11]–[19]. In the present study, we restrict our interest to the ferret model, especially its use in examining respiratory transmission, for consistency and clarity both in theory and biology. Among the total of 12 studies, nine investigated H1N1 viruses including two on 1918–19 pandemic viruses and seven on 2009 pandemic viruses. Three studies investigated the transmissibility of H5N1 viruses, two on H3N2 viruses and one on H2N2 viruses. In principle, those studies share the experimental design (i.e. inoculation of one ferret in a cage and exposure of the other ferret in an adjacent cage), but the details have been variable. The air flow (e.g. direction and air exchange rate) has not been strictly regulated by common rules, and viral dose for inoculation have not been identical among these studies, and thus, various differences in experimental designs prohibit pooling of data from differently designed studies.

**Table 1:** Study characteristics of the published one-to-one animal transmission experiment of influenza A viruses.

	Year of publication	Subtype	Infected animals in control group (k/n)†	Infected animals in comparison group (k/n)†	Judgment^‡^	Study objective(s)
Maines et al. [11]	2006	H3N2 & H5N1	3/3	0/3	Different	To identify the importance of virus internal protein genes in regulating transmissibility
Tumpey et al. [12]	2007	H1N1	3/3	0/3	Different	To show that only a modest change in the 1918 influenza hemagglutinin receptor binding site alters the transmissibility
van Hoeven et al. [8]	2009	H1N1	3/3	0/3	Different	To identify genetic determinants that govern airborne transmission among 1918-avian H1N1 ifnluenza reassortant viruses
Munster et al. [13]	2009	H1N1	4/4	4/4	Similar	To study aerosol transmission of the pandemic 2009 A(H1N1) influenza virus as compared with a seasonal 2007 A(H1N1) virus
Maines et al. [14]	2009	H1N1	3/3	2/3	Different	To compare the transmissibility of H1N1-2009 against a seasonal H1N1 virus through respiratory droplets
Pappas et al. [15]	2010	H2N2	3/3	0/3	Different	To examine the transmissibility of human H2N2 viruses isolated during the 1957/58 pandemic
Kiso et al. [16]	2010	H1N1	3/3	3/3	Similar	To compare the transmissibility between oseltamivir-resistant and sensitive H1N1-2009.
Van Doremalen et al. [17]	2011	H1N1	3/3	3/3	Similar	To investigate the effect of residue 227 in hemagglutinin on cell tropism and transmission of pH1N1 2009.
Koster et al. [18]	2012	H1N1	4/4	4/4	Similar	To develop a method to detect exhaled viral aerosol transmission between unanesthetized infected and susceptible ferrets.
Pearce et al. [19]	2012	H3N2	3/3	3/3	Similar	To analyze the transmissibility of four A(H3N2)v influenza viruses isolated from humans in 2009, 2010 and 2011.
Herfst et al. [5]	2012	H5N1 & H1N1	0/2	3/4	Different	To address the concern that the virus could acquire the ability of airborne transmission under natural conditions
Imai et al. [3]	2012	H5N1 & H1N1	0/3	4/6	Different	To assess the molecular changes in HA that would allow a virus possessing subtype H5 HA to be transmissible

† *n* represents the sample size, i.e. the number of pairs of a single infected animal and a susceptible animal. *k* represents the number of pairs with successful transmission. The numbers among control group are the highest reported frequency of infected animals in control group, while the numbers among comparison group show the lowest reported frequency of infection. ^‡^Judgment corresponds to the interpretation of difference in transmissibility.

With regard to the sample size (i.e. the number of pairs for each virus), all studies used small numbers of ferrets, with most choosing 3 pairs of infectious/susceptible ferrets in each group. There were two studies that used 4 pairs. Two studies used different pair numbers between two groups, i.e., one study used 2 pairs for the control and 4 pairs for the comparison group, and the other study used 3 pairs for the control and 6 pairs for the comparison group. The results (i.e. the number of pairs with successful transmission) shown in Table 1 represent the highest and lowest reported numbers among the all combinations of two viruses, and the judgment of difference (or similarity) was drawn in the original publications based on the corresponding results. The primary objective of the original studies was either to identify molecular mechanisms (e.g. specific viral gene, amino acid or protein) governing the transmissibility (*n* = 5) or to quantify or compare the capacity of aerosol transmissibility (*n* = 7).

### Stochastic general epidemic model

To allow comparison of the transmissibility, here we express the result from one-to-one transmission experiment (i.e. the proportion of pairs with transmission) as a function of *R*_0_. First of all, we adopt an assumption that each pair is independent of other pairs, including no air-exchange between pairs. Let *p*_s,i_(*t*) be the conditional probability of observing *s* susceptible and *i* infected ferrets at time *t* given the initial condition of susceptible and infected ferrets (*s*_0_, *i*_0_) at time 0, i.e.,
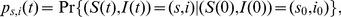
(1)then the so-called “stochastic general epidemic” model is described by the following differential-difference equation:
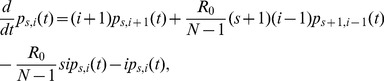
(2)where N is the total population size (N = 2 in the case of one-to-one experiment) and t represents the multiple of the mean infectious period (i.e. the time unit is normalized by the mean infectious period). Here it should be noted that the mass action part has been scaled by (N−1), and not by N, because of small population size that requires us to precisely consider the impact of N on the incidence term. That is, in the case of small N, the transient risk of infection should be proportional to I(t)/(N−1) which can be exemplarily understood for N = 2 (i.e. if we use I(t)/N for calculating the incidence, it would indicate erroneously that the half of infectious individuals I(t)/2 would contribute to the transmission). Since the initial condition gives p_s0_,_i0_(0) = 1, the probability of successful transmission by infinite time, q, is computed by p_0,0_(∞) [20]–[22] and the solution is



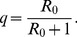
(3)Note that the analytical solution of *q* for small *N* is *q* = *R*_0_/(*R*_0_+*N*−1) which is different from what has been previously discussed [22]. Since the one-to-one transmission experiment handles the binary outcome (i.e. success or failure of transmission), the probability of transmission is computed by employing a binomial distribution. That is, for *n* independent pairs of one-to-one transmission experiments, the probability of observing *k* pairs with successful transmission is
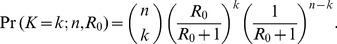
(4)


The maximum likelihood estimator of *R*_0_ based on the observed average frequency of successful transmission, *k*/*n*, is given by equating *R*_0_/(*R*_0_+1) = *k*/*n* which yields.
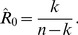
(5)


The 100(1-*α*)% confidence interval of *R*_0_ is calculated from the solution of *R*_0,CI_ = *x*/(*n*-*x*) in which *x* satisfies
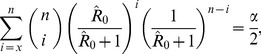
(6)for the upper bound and
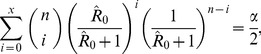
(7)for the lower bound, except that the lower bound is 0 when x = 0 and the upper bound is infinity when x = n. In these exceptional circumstances, the upper bound for x = 0 is calculated as R_0,upper_ = ((α/2)^(1/n)^-1)/(α/2)^(1/n)^ and the lower bound for x = n is calculated as R_0,lower_ = (α/2)^(1/n)^/(1-(α/2)^(1/n)^) as can be derived from the binomial distribution [23]. In addition to the final size discussed above, statistical consideration of transient state has been given elsewhere [24].

### Hypothesis testing

We subsequently consider the difference in the transmissibility in published experimental studies in two different ways, because the null hypothesis has not necessarily been mentioned in the original articles in Table 1. Let *R*_0,ref_ be a specified reference value of the basic reproduction number. The first possible way to compare the transmissibility is to regard the result from each virus as one-sample comparison, which may be the case when *R*_0,ref_ of control virus can be assumed known (e.g. pre-determined) from published studies and so on. In this scenario, we compare *R*_0_ against *R*_0,ref_, i.e.
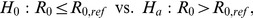
(8)which may sometimes be intended to support the notion that some key molecular structure helped to acquire substantial transmissibility for a specific virus (e.g. by setting *R*_0,ref_ = 1 or *R*_0,ref_ = 0). It should be noted that *R*_0_ depends on experimental design (air change rate per hour, air flow direction, etc) and is not comparable between differently designed experiments. Using the relationship (5), the issue of comparing transmissibility is replaced by one-sample comparison of a binomial proportion. The p-value for testing (8) given that *k* or more pairs resulted in infection is computed by
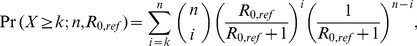
(9)which rejects H_0_ if less than or equal to α = 0.05. Demonstrating R_0_>R_0,ref_ = 0 would help researchers to demonstrate the capacity of a ferret infected with a virus to “cause” (any number of) secondary transmission. Demonstrating R_0_>R_0,ref_ = 1 is particularly useful in practice, because satisfying this condition indicates that, at least under experimental conditions, a single primary case can on average generate one or more secondary cases through the respiratory route (and thus, is regarded as possessing a substantial transmission potential to cause an epidemic only through that particular mode of transmission). Of course, rejecting H_0_: R_0_≥1 can be achieved similarly by calculating Pr(X<k; n,R_0,ref_ = 1)≤0.05. The power for (8) is given by
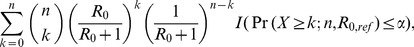
(10)where *I*(.) is the indicator function.

The second way to test the transmissibility is to consider the two virus groups within the same study in Table 1 as the comparison of two binomial proportions, *q*_1_ and *q*_2_ (or equivalently the comparison of two basic reproduction numbers estimated for respective viruses, *R*_0,1_ and *R*_0,2_) under the hypotheses
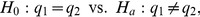
(11)(or *H*_0_: *R*_0,1_ = *R*_0,2_) and the implementation is exactly the same as two-tailed exact test for two samples that has been already discussed elsewhere [25]. It should be noted that differing number of pairs between two virus groups can be easily addressed by varying sample sizes in the exact test. In both one-sample and two-sample cases, the sample size estimation would have to be made directly from the binomial distribution (e.g. from (10) with a desired power). However, as an alternative, the power calculation could rest on a modified Wald test statistic, i.e., the well-known score confidence interval proposed by Agresti and Coull [26], [27], [28].

For numerical illustrations, we consider the p-value and power for all possible patterns of final size for the number of pairs, *n* = 3, 4 and 5 for both one-sample and two-sample comparisons. These numbers of pairs are specifically considered, because 3 pairs have been conventionally adopted, and we anticipate that 6 or more pairs may not be logistically very feasible for testing many types of influenza virus at present. A one-sample comparison is made by a one-tailed Fisher's exact test, while a two-sample comparison rests on a two-tailed test. While restricting our consideration to *n* = 3, 4 and 5, we also examine the p-value for one-sample test with varying reference values of the basic reproduction number and the number of pairs (from 1 to 10), especially in the case we have *k* = *n* (i.e. all pairs resulted in infection) or *k* = *n*−1 which are frequently the case in published experimental studies.

## Results

### One-sample comparison

**Figure 1:**
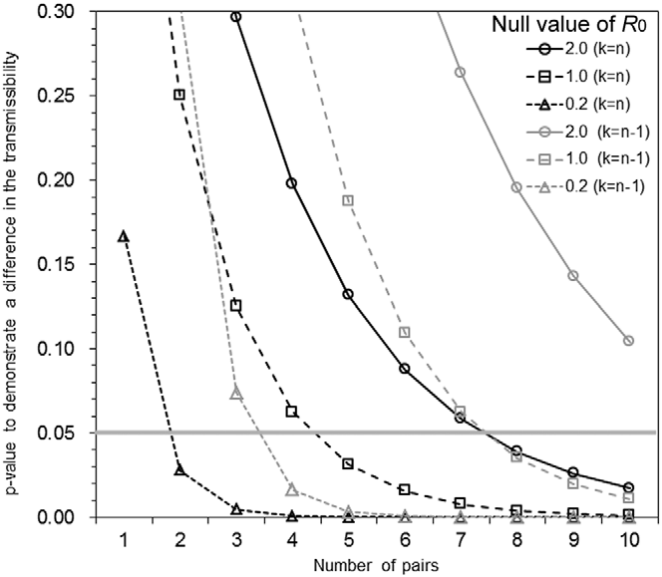
The p-value to demonstrate the significant difference in the transmissibility based on fully or nearly fully successful one-to-one transmission experiment. The p-values are shown to indicate the significance level at which the estimated basic reproduction number is significantly greater than the null value (*R*_0,null_ are set to be 0.2, 1.0 and 2.0 with the null hypothesis, *H*_0_: *R*_0_≤*R*_0,null_) given that all pairs resulted in infection (black lines; *n* = *k* where *n* and *k* are the numbers of pairs and infected pairs, respectively) or all pairs minus 1 resulted in infection (grey lines; *n* = *k*+1). The hypothesis testing is based on one-sample comparison using the one-tailed Fisher's exact test. The horizontal grey bold line represents the significance level at 0.05.

Table 2 shows the p-value and power for one-sample comparisons given that the number of pairs was 3, 4 or 5. Even when all pairs result in infection during 3 or 4 pair study, the experiment cannot indicate that the *R*_0_ is significantly greater than 1. Only when we have a result of 5/5, the difference can be stated as significantly greater than *R*_0_ of 1. Moreover, in the cases all pairs result in infection, one can quantitatively examine only the lower bound of *R*_0_, and the expected value and the upper bound of *R*_0_ are calculated as infinite. Figure 1 shows the p-value with different numbers of pairs in the case that all pairs are infected (i.e. *k* = *n*) or all pairs minus 1 resulted in infection (i.e. *k* = *n*−1). When the reference value of *R*_0_ is as large as 2, three pairs are not large enough to demonstrate *R*_0_>2 at a significant level *α* = 0.05, and one may need at least 8 pairs and all the eight pairs need to be infected. At a stricter threshold (e.g. *α* = 0.01), seven or more pairs would be required to reject *R*_0,null_ = 1. In the case of *k* = *n*−1, even ten pairs would not be enough to demonstrate that *R*_0_>2 at *α* = 0.05 given *k* = *n*−1.

**Table 2:** One-tailed test results of the basic reproduction number based on one-to-one transmission experiment.

Number of pairs	Final size	*R*_0_ †	(95% CI^‡^)	p-value to demonstrate *R*_0_>1	power to demonstrate *R*_0_>1	power to demonstrate *R*_0_>0	p-value to demonstrate *R*_0_<1
3	0	0.0	(0, 4.8)	1.00	0.00	1.00	1.00
	1	0.5	(0, ∞)	0.88	0.00	1.00	0.74
	2	2.0	(0, ∞)	0.50	0.00	1.00	0.70
	3	∞	(0.8, ∞)	0.13	0.00	1.00	1.00
4	0	0.0	(0, 3.0)	1.00	0.00	0.00	1.00
	1	0.3	(0, 6.0)	0.94	0.00	0.68	0.74
	2	1.0	(0, ∞)	0.69	0.00	0.94	0.69
	3	3.0	(0.7, ∞)	0.31	0.00	0.99	0.68
	4	∞	(1.3, ∞)	0.06	0.00	1.00	1.00
5	0	0.0	(0, 2.2)	1.00	0.00	0.00	1.00
	1	0.3	(0, 3.0)	0.97	0.00	0.67	0.74
	2	0.7	(0, 8.0)	0.81	0.00	0.92	0.68
	3	1.5	(0.5, ∞)	0.50	0.08	0.99	0.66
	4	4.0	(1.3, ∞)	0.19	0.33	1.00	0.67
	5	∞	(1.8, ∞)	0.03^¶^	1.00	1.00	1.00

† The basic reproduction number, estimated from the one-to-one transmission experiment. ^‡^CI, confidence intervals.^¶^ *R*_0_ is significantly greater than 1 by one-sample Fisher's exact test.

Also, when one pair escapes infection (i.e. *k* = *n*−1), Figure 1 and Table 2 consistently suggest that a five-pair or smaller study cannot help judge if *R*_0_ is significantly greater than 1. The results 2/3, 3/4 or 4/5 does not indicate significant difference from *R*_0_ = 1. All other combinations in Table 2 cannot determine if *R*_0_ is significantly greater than 1, and more importantly, either lower or upper 95% confidence interval of estimated *R*_0_ for these combinations always takes an extreme value (i.e. either lower bound being 0 or upper bound being infinite).

Provided that a transmission study intends to demonstrate *R*_0_>0, the presence of at least one successful transmission (i.e. any *k*/*n* except for *k* = 0) can yield a significant result with p<0.01. However, it should be remembered that power is not substantial for *k*/*n* = 1/4 and 1/5 (Table 2). When one intends to demonstrate that the transmission potential is less than 1, the examined total sample sizes are not enough to argue significant differences (Table 2). That is, given the number of pairs is 3, 4 or 5, it is more feasible to show that *R*_0_>1 than demonstrating *R*_0_<1.

### Two-sample comparison

Table 3 summarizes the p-value and power for two-sample comparisons given that the number of pairs was 3, 4 or 5. Given three pairs for each sample, it is impossible to demonstrate any significant difference between two sample groups. In the case of four pairs, only a combination of 0/4 and 4/4 can indicate a significant difference. Given five pairs, three combinations (i.e. 0/5 vs 5/5, 1/5 vs 5/5 or 0/5 vs 4/5) could suggest significant difference in the transmission potential.

**Table 3:** Two-tailed comparison of the basic reproduction numbers based on one-to-one transmission experiment (*H*_0_: *R*_0_ = *R*_0,null_ †).

		0		1		2		3		4		5	
Number of pairs	Final size of comparison group	p-value	power	p-value	power	p-value	power	p-value	power	p-value	power	p-value	power
3	0	1.00	0.00	1.00	0.04	0.40	0.30	0.10	1.00				
	1	1.00	0.04	1.00	0.00	1.00	0.09	0.40	0.30				
	2	0.40	0.30	1.00	0.09	1.00	0.00	1.00	0.04				
	3	0.10	1.00	0.40	0.30	1.00	0.04	1.00	0.00				
4	0	1.00	0.00	1.00	0.05	0.43	0.31	0.14	0.74	0.03^¶^	1.00		
	1	1.00	0.05	1.00	0.04	1.00	0.13	0.49	0.37	0.14	0.74		
	2	0.43	0.31	1.00	0.13	1.00	0.07	1.00	0.13	0.43	0.31		
	3	0.14	0.74	0.49	0.37	1.00	0.13	1.00	0.04	1.00	0.05		
	4	0.03^¶^	1.00	0.14	0.74	0.43	0.31	1.00	0.05	1.00	0.00		
5	0	1.00	0.00	1.00	0.01	0.44	0.09	0.17	0.34	0.05^¶^	0.74	0.01^¶^	1.00
	1	1.00	0.01	1.00	0.01	1.00	0.03	0.52	0.14	0.29	0.38	0.05^¶^	0.74
	2	0.44	0.09	1.00	0.03	1.00	0.02	1.00	0.05	0.52	0.14	0.17	0.34
	3	0.17	0.34	0.52	0.14	1.00	0.05	1.00	0.02	1.00	0.03	0.44	0.09
	4	0.05^¶^	0.74	0.21	0.38	0.52	0.14	1.00	0.03	1.00	0.01	1.00	0.01
	5	0.01^¶^	1.00	0.05^¶^	0.74	0.17	0.34	0.44	0.09	1.00	0.01	1.00	0.00

† *R*_0,null_, the basic reproduction number to be used in null hypothesis. ^¶^Estimated transmissibility from one-to-one transmission experiment is significantly different from *R*_0,null_ by two-sample Fisher's exact test.

## Discussion

The present study discussed the sample size considerations for one-to-one experimental studies of the transmission of influenza A viruses. Employing the stochastic general epidemic model, *R*_0_ was derived from the final state of an epidemic [29], [30], [31], and its relevance to the probability of successful transmission during the one-to-one trial was explained. Three findings are particularly notable. First, *k*/*n* = 3/3 and 4/4 are not indicative of significant excess of *R*_0_ from 1 in one-sample comparison. At least, five pairs would be required to demonstrate significant difference in the one-sample comparison. Second, *n* = 3 is not enough to show any significant difference in two-sample comparisons. Third, *k* = *n* can yield the significant difference when *n* = 5 or greater in one-sample comparison, but one has to remember that the expected value and the upper confidence interval of *R*_0_ would be calculated as infinite for small *n*. That is, while the experiment may be able to show significant difference from reference value, *k* = *n* can inform only the lower bound of *R*_0_. With the very limited sample sizes such as *n* = 3, 4 or 5, it is always the case that either lower or upper 95% confidence interval takes an extreme value (i.e. lower  = 0 or upper  = ∞). Keeping these points in mind, one can plan the one-to-one transmission study with reference to our computed results in Tables 2 and 3, while relating the observed proportion of infected pairs to *R*_0_, an interpretable epidemiological measure of transmissibility.

The most important caveat in the present study in relation to the common practice is that *n* = 3 is not enough to show a significant difference as well as *R*_0_>1 for one-sample comparison, while it can demonstrate *R*_0_>0. Moreover, comparing a group with *n* = 3 against a reference group with the same sample size does not allow researchers to demonstrate any significant difference in the transmissibility between two sample groups. If two samples have to be compared, *n* = 4 would be regarded as minimum, and moreover, *k* = *n* for *n* = 4 and *k* = *n* or *k* = *n*−1 for *n* = 5, respectively, would have to be required along with the absence of infected pairs in the control group. To interpret some results of the published studies in Table 1 which concluded difference in the transmissibility between two viruses, one sample interpretation may better be adopted for each virus against *R*_0,null_ = 0 (rather than comparing two sample groups). Over-interpretation of results without significant difference should be avoided.

It should be noted that demonstrating similar transmissibility between two groups is even more difficult in this context for two reasons. First, the sample size (i.e. the number of pairs) is very limited, and thus, it is too frequent that we do not observe any significant difference between two sample groups (Table 3). Second, demonstrating similarity must be distinguished from showing the absence of a significant difference, especially when the sample sizes are very limited. Demonstrating similarity in transmissibility would likely require much larger sample sizes, as seen in noninferiority and equivalence randomized trials [32]. Similarly, demonstrating the absence of substantial transmissibility in a single sample group is also a difficult task. As was shown in Table 2, showing *R*_0_<1 cannot be achieved by *n* = 3, 4 or 5.

Two limitations should be noted. First, every transmission experiment study examines not only the frequency of successful transmissions but also other factors including mortality, weight loss, patterns in virus shedding, clinical signs and symptoms, behavioral changes and so on. Thus, although the quantification of transmissibility should strictly adhere to the frequency of transmission events, the interpretation of adaptation, pathogenicity and infectiousness (e.g. in the sense of virus replications in infected hosts) should be judged from multiple results. Moreover, study objectives of transmission experiment may not necessarily be to demonstrate differential transmissibility (e.g. may only be to prove that pre-symptomatic transmission can occur [33]). In this regard, the present study has focused only on a single aspect of experimental findings. Second, we adopted an independence assumption between pairs which may not be strictly the case in all published studies. If the transmission studies handled infectious virus with substantially high aerosol transmission potential, this assumption is violated. However, explicitly addressing this point cannot be achieved by employing a simple general stochastic epidemic model, and rather, would require much more complex modeling analysis. Resolving second problem (of dependence between pairs) by mathematical modeling are our forthcoming future studies.

It is important that the researchers can calculate the most appropriate sample size (with or without a reference sample group) depending on study objectives. Sample size rationale should be formulated in the future including other types of transmission experiment design, such as exposing multiple animals to multiple infectious animals, or exposing two animals in two different manners (e.g. with and without direct contact). As the translation of the proportion of infected pairs into *R*_0_ can permit object-oriented experimental design, the objectives, design and findings of transmission experiment studies have to be reviewed and the corresponding sample size rationale should be discussed. The present study could be regarded as a starting point to extensively consider hypothesis testing of animal transmission experiment results using epidemiologically well-defined parameters.
